# Increased frequencies of highly activated regulatory T cells skewed to a T helper 1-like phenotype with reduced suppressive capacity in dengue patients

**DOI:** 10.1128/mbio.00063-24

**Published:** 2024-05-16

**Authors:** Sotheary Sann, Borita Heng, Hoa Thi My Vo, Rebeca Arroyo Hornero, Sokchea Lay, Sopheak Sorn, Sreymom Ken, Tey Putita Ou, Denis Laurent, Chantana Yay, Sowath Ly, Philippe Dussart, Veasna Duong, Anavaj Sakuntabhai, Markus Kleinewietfeld, Tineke Cantaert

**Affiliations:** 1 Immunology Unit, Institut Pasteur du Cambodge, Pasteur Network, Phnom Penh, Cambodia; 2 VIB Laboratory of Translational Immunomodulation, Hasselt University, Diepenbeek, Belgium; 3 Department of Immunology, Hasselt University, Diepenbeek, Belgium; 4 University Multiple Sclerosis Center, Hasselt University, Diepenbeek, Belgium; 5 Epidemiology and Public Health Unit, Institut Pasteur du Cambodge, Pasteur Network, Phnom Penh, Cambodia; 6 Virology Unit, Institut Pasteur du Cambodge, Pasteur Network, Phnom Penh, Cambodia; 7 Kantha Bopha Children's Hospital, Phnom Penh, Cambodia; 8 Jayavarman VII Hospital, Siem Reap, Cambodia; 9 Department of Global Health, Ecology and Emergence of Arthropod-borne Pathogens, Institut Pasteur, Université de Paris, Paris, France; 10 Université de Paris-Cité, CNRS UMR 2000, Paris, France; 11 Institut National de Recherche pour l'Agriculture, l'Alimentation et l'Environnement (INRAE) USC 1510, Paris, France; University of Hong Kong, Pokfulam, Hong Kong Nil, Hong Kong

**Keywords:** dengue virus, regulatory T cells, FOXP3, severe dengue disease

## Abstract

The pathogenesis of dengue involves a complex interplay between the viral factor and the host immune response. A mismatch between the infecting serotype and the adaptive memory response is hypothesized to lead to exacerbated immune responses resulting in severe dengue. Here, we aim to define in detail the phenotype and function of different regulatory T cell (Treg) subsets and their association with disease severity in a cohort of acute dengue virus (DENV)-infected Cambodian children. Treg frequencies and proliferation of Tregs are increased in dengue patients compared to age-matched controls. Tregs from dengue patients are skewed to a Th1-type Treg phenotype. Interestingly, Tregs from severe dengue patients produce more interleukin-10 after *in vitro* stimulation compared to Tregs from classical dengue fever patients. Functionally, Tregs from dengue patients have reduced suppressive capacity, irrespective of disease severity. Taken together, these data suggest that even though Treg frequencies are increased in the blood of acute DENV-infected patients, Tregs fail to resolve inflammation and thereby could contribute to the immunopathology of dengue.

**IMPORTANCE:**

According to the World Health Organization, dengue is the fastest-spreading, epidemic-prone infectious disease. The extent of dengue virus infections increased over the years, mainly driven by globalization—including travel and trade—and environmental changes. Dengue is an immunopathology caused by an imbalanced immune response to a secondary heterotypic infection. As regulatory T cells (Tregs) are essential in maintaining immune homeostasis and dampening excessive immune activation, this study addressed the role of Tregs in dengue immunopathology. We show that Tregs from dengue patients are highly activated, skewed to a Th1-like Treg phenotype and less suppressive compared to healthy donor Tregs. Our data suggest that Tregs fail to resolve ongoing inflammation during dengue infection and hence contribute to the immunopathology of severe dengue disease. These data clarify the role of Tregs in dengue immunopathogenesis, emphasizing the need to develop T cell-based vaccines for dengue.

## INTRODUCTION

Half of the world’s population lives in areas where dengue virus (DENV) is transmitted, causing an estimated 400 million new infections every year (1, 2). The extent of DENV infections increased over the years, mainly driven by globalization—including travel and trade—and environmental changes (3). Although DENV infection mostly results in asymptomatic or mild disease, it is estimated that about 10%–20% of infections can lead to hospitalization (2, 4). Dengue can be subdivided in either classical dengue fever (DF) or more severe disease, including dengue hemorrhagic fever (DHF) and dengue shock syndrome (DSS) (5, 6). DHF and DSS are characterized by thrombocytopenia and vascular leakage, resulting in significant morbidity and mortality mainly in young children (5, 6). Patients with plasma leakage have increased levels of soluble mediators such as proinflammatory and vasoactive cytokines which are elevated before and at the time of plasma leakage (7–11). Four serotypes of DENV are known and circulate concomitantly around the globe, and individuals living in endemic regions experience multiple infections during their lifetime (3). Interestingly, individuals undergoing a secondary heterotypic infection have a higher risk to develop severe dengue disease, indicating that the adaptive immune response to DENV has both protective and detrimental aspects (1, 9, 12). A mismatch between the infecting serotype and the memory adaptive immunity is proposed to result in suboptimal immune responses leading to severe dengue (DHF/DSS) (13). Here, reactivation of memory T cells with lower specificity for the secondary infecting serotype might result in less efficient viral clearance (14). On the other hand, more recent research has shown protective roles for T cells during DENV infection (15). For example, we have shown that asymptomatic infected patients show an increased activation of the CD4^+^ and CD8^+^ T-cell compartment compared to hospitalized dengue patients (9). In addition, frequency and functionality of CD4 effector memory T cells expressing CD45 seem to be associated with protective immunity (16, 17). A higher magnitude and polyfunctional CD8^+^ T-cell response has been observed for human leukocyte antigen (HLA) alleles associated with decreased susceptibility to severe disease, suggesting a potential protective role for CD8^+^ T cells (18).

Regulatory T cells (Tregs) are essential in maintaining immune homeostasis and peripheral tolerance (19–21). They are characterized by a high expression of the IL-2 receptor α-chain (CD25), the expression of the transcription factor FOXP3, and decreased expression of the IL-7 receptor α-chain (CD127) (19, 20). Tregs have the ability to suppress and neutralize responses of the innate and adaptive immune system by various mechanisms like secretion of anti-inflammatory cytokines such as interleukin (IL)-10, via cell–cell contact-dependent mechanisms, for example, via ligation of cytotoxic T lymphocyte-associated protein 4 (CTLA-4) or via depletion of removal of the danger signal adenosine triphosphate (ATP) or production of anti-inflammatory adenosine via enzymatic activity of CD39 and CD73 (22, 23).

Similar to CD4^+^ T helper (Th) subsets, Treg cells can adopt features of Th cell subsets, where the chemokine receptor expression pattern in Th-like Tregs is similar as in conventional Th subsets (24, 25). Each Th-like Treg population shows unique patterns of pro-and anti-inflammatory cytokine production and expression of lineage-specific transcription factors and is differentially activated by antigens associated with either Th1 or Th17 responses. Next to FOXP3, Helios is an important transcription factor in Treg biology, whose presence might be used to define naturally occurring, thymic-derived Tregs, although it seems that Helios can also be upregulated in activated and proliferating Tregs (26–28).

In the context of infectious diseases, Tregs have both beneficial and harmful effects: while they dampen excessive immune activation, they also can suppress beneficial antigen-specific immune responses (29, 30). During the acute phase of infection, increased frequencies of Tregs in the circulation of dengue-infected patients compared to healthy controls have been observed (31–33). However, it remains unclear if there is an association between specific Treg subsets and Treg functions with disease outcome (32, 34–38). Given the current increased understanding of different Treg subsets and their functional properties, we aimed to define in detail the phenotype and function of different Treg subsets and their association with disease severity in a cohort of acute DENV-infected, Cambodian children. We observed that Tregs obtained from DENV-infected children are skewed to a pro-inflammatory Th1-like Treg phenotype with increased production of cytokines after stimulation. In line with this, Tregs from DENV patients have reduced suppressive capacity *in vitro*. Interestingly, Tregs from severe dengue patients produced higher amounts of both IL-10 and interferon gamma (IFN-γ), even though the expression of functional markers did not differ from Tregs from age-matched healthy donors (HDs), irrespective of disease severity. Taken together, these data suggest that even though Treg frequencies are increased in the blood of acute DENV-infected patients, Tregs fail to resolve inflammation and thereby could critically contribute to the immunopathology of dengue.

## MATERIALS AND METHODS

### HD and dengue patient recruitment

Blood samples were collected from hospitalized children (≥2 years old) with a dengue-like syndrome, who were admitted at Kantha Bopha hospital in Phnom Penh, Jayavarman VII Hospital in Siem Reap, and three hospitals in Kampong Thom province, Cambodia. The time point of blood sample collection was less than 96 h after fever onset. Dengue-positive patients were classified based on the World Health Organization (WHO) 1997 criteria upon hospital discharge into DF, DHF, and DSS (5). The age-matched HDs were selected among participants of a household investigation in Kampong Thom province, as described before (39). For the *in vitro* infection experiments, adult HDs were recruited from volunteers who presented at the International Vaccination Centre, Institut Pasteur du Cambodge. Demographic data and clinical characteristics of HD and dengue patient groups included in this study are described in Table 1.

**TABLE 1:** Demographic data and clinical characteristics of the studies cohort^*a*^

Cohort	DF	DHF/DSS	HD (children)	HD (adults)
	*N* = 26	*N* = 23	*N* = 28	*N* = 10
Gender				
Female	15 (58%)	11 (48%)	14 (50%)	2 (20%)
Male	11 (42%)	12 (52%)	14 (50%)	8 (80%)
Age				
Mean (SD)	10.1 (3.3)	10.2 (2.7)	8.9 (3.8)	31.8 (5.7)
Hematocrit (%)				
Mean (SD)	38.9 (5.5)	44.7 (5.7)	N/A	N/A
Platelets (×10^9^/L)				
Mean (SD)	111.8 (39.3)	82.6 (33.6)	N/A	N/A
Day of fever				
Mean (SD)	2.8 (1.2)	3.8 (1.2)	N/A	N/A
Serotype				
DENV1	14	10	N/A	N/A
DENV2	10	13	N/A	N/A
DENV3	1	0	N/A	N/A
DENV4	1	0	N/A	N/A
Immune status				
Primary	1	1	N/A	N/A
Secondary	23	20	N/A	N/A
Undetermined	2	2	N/A	N/A
NS1 rapid test +	13	12	N/A	N/A
DENV RT-qPCR +	26	23	N/A	N/A
Viral load (×10^2^ copies/mL)				
Mean (SD)	2.43 (7.64)	2.02 (5.93)	N/A	N/A

^*a*^ N/A: not available. HD: healthy donor. SD: standard deviation. DF: dengue fever. DHF: dengue hemorrhagic fever. DSS: dengue shock syndrome. NS1: non-structural protein 1.

### Laboratory diagnosis

Plasma samples from patients and HDs were tested for the presence of DENV by real-time reverse transcription polymerase chain reaction (RT-qPCR) at Institut Pasteur du Cambodge as described before (40). Rapid diagnosis tests (SD Bioline Dengue Duo kits, Standard Diagnostics, Abbott, USA) were used to detect the presence of DENV-non-structural protein 1 (NS1), anti-IgM, and anti-IgG in patient plasma. Anti-DENV IgM in patient serum was measured using an IgM-capture ELISA as described (41). Patients were identified as having primary/secondary DENV infection using a hemagglutination inhibition test in line with the WHO criteria (5). Laboratory results and patient classifications are shown in Table 1. Acute DENV-infected cases are defined as patients with less than 96-h onset of fever and a positive DENV RT-qPCR. Peripheral blood mononuclear cells (PBMCs) were isolated from human blood samples by using Ficoll–Histopaque (Sigma-Aldrich) density gradient centrifugation. The cells were resuspended in 10% of dimethyl sulfoxide (Sigma-Aldrich) and 90% of fetal bovine serum (FBS) (Thermo Fisher Scientific) and stored in liquid nitrogen until analysis.

### *Ex vivo* phenotyping of Treg subsets

The cryopreserved cells were thawed into warm RPMI (Sigma-Aldrich) added with 10% FBS and counted for flow cytometry analysis. Three flow cytometry panels were designed to characterize Treg activation state, migration, and functional markers (Tables S1 and S2). All phenotypic analysis was done in Cambodia, where at the time of analysis, only a FACSCanto II equipment was available. Therefore, combinations of markers and panels are restricted.

After counting, at least 500,000 viable cells were used per panel. First, the cells were stained with Zombie Aqua fixable viability kit (BioLegend) to determine live/dead cells. Cells were stained for 30 min on ice with surface markers as described in Table S1. After washing, the cells were fixed and permeabilized with True-Nuclear Transcription Factor Buffer Set (BioLegend) per manufacturer’s instruction followed by intracellular staining with anti-FOXP3 and CTLA-4 antibodies for 30 min on ice. All surface and intracellular antibodies were purchased from BioLegend (Table S3). The cells were analyzed with BD FACSCanto II (BD Biosciences). For the phenotyping experiments (Table S1, panels 1–3), a total of 17 age-matched HDs and 35 pediatric dengue cases were included

### Intracellular cytokine measurement

Starting from 500,000 viable cells, PBMCs were stimulated with phorbol myristate acetate (PMA) (Sigma-Aldrich) and ionomycin (Sigma-Aldrich), and cytokine secretion was inhibited using Monensin (BioLegend) for 6 h at 37°C. The cells then were stained for live/dead cells with Zombie Aqua (BioLegend) followed by surface staining with surface markers as described in Table S2 for 30 min on ice. The cells were subsequently treated with True Nuclear Transcription Factor Buffer Set (BioLegend) per manufacturer’s guidelines before performing intracellular staining with anti-FOXP3, Helios, Ki-67, IFN-γ, IL-10, and IL-17A antibodies for 30 min on ice. After washing, the cells were read with BD FACSCanto II. For this experiment, a total of 15 age-matched HD and 34 pediatric dengue cases were included. Of the HD, 14 overlapped with HD included in panels 1–3. Of the pediatric dengue cases, 29 overlapped with patients included in panels 1–3. This was due to a limited amount of cells available for some of the patients.

### *In vitro* suppression assay

CD4^+^ T cells were enriched from fresh isolated PBMCs by using negative selection CD4^+^ T-cell microbeads (Miltenyi Biotec) following manufacturer’s protocol. The untouched CD4^+^ T cells were stained with anti-CD4, CD25, and CD127 antibodies. Two populations of cells: CD4^+^CD25^+^CD127 low Treg and CD4^+^CD25^−^ responder T cells (Tresp) were sorted by BD FACSAria (BD Biosciences). For proliferation tracking, isolated Tresp were subsequently stained with carboxyfluorescein diacetate succinimidyl ester (BioLegend) as per manufacturer’s guideline. The cells were counted by Countless II FL Automated Cell Counter. As a positive control for the assay, 10,000 Tresp cells were cultured with 10,000 Treg suppressive inspector beads (CD2/CD3/CD28 containing beads) (Miltenyi Biotec). Suppressive capacity of Tregs was evaluated using a co-culture of 5,000 Treg cells and 5,000 Tresp cells in the presence of 10,000 Treg suppressive inspector beads. Cells were cultured in round-bottom well plates in complete RPMI [RPMI supplemented with 5% human serum (Sigma-Aldrich), penicillin (100 U/mL, Thermo Fisher Scientific), streptomycin (100 µg/mL, Thermo Fisher Scientific), and L-glutamine (2 mM, Invitrogen)] and incubated at 37°C with 5% CO_2_. At day 5 of culture, the cells were stained with Zombie Aqua (BioLegend) according to the manufacturer instructions for viability and then were read with BD FACSAria Fusion (BD Bioscience). For this experiment, 10 pediatric dengue cases and 10 age-matched healthy controls were used, which were different donors from the *ex vivo* phenotyping experiments due to the low amounts of PBMC available per patient.

### *In vitro* infection for Treg-specific mechanism

Fresh PBMCs from adult HDs were infected with DENV-2 New Guinea C (GenBank: AF038403). Production of viral stocks has been previously described (42). In detail, 500,000 viable cells were used in the round-bottom plate for the infection at multiplicity of infection 0.1 and 1 (MOI 0.1 and 1) for 90 min at 37°C. Supernatants from non-infected cells were used for uninfected control to eliminate the background from non-specific stimulation by foreign proteins or antigens in virus culture. After infection, the cells were washed and resuspended in 200 µL of complete RPMI and cultured at 37°C for 3 days. DENV infection was measured at Day 1. The cells were stained for live/dead cells with Zombie Aqua (BioLegend) followed by surface marker staining for anti-CD3, CD14, and CD56 (Table S3) for 30 min on ice. The cells were subsequently treated with True Nuclear Transcription Factor Buffer Set (BioLegend) per manufacturer’s guidelines before performing intracellular staining with anti-DENV E protein antibody (clone 4G2, ATCC HB-112) labeled with Alexa Fluor 488. At day 3, the cells were collected for Treg phenotyping and stained with Zombie Aqua (BioLegend) followed by antibodies against surface markers: anti-CD3, CD4, CD25, CD127, CD39, CD45RA, HLA-DR, CXCR3, CCR4, CCR6, inducible T-cell costimulator (ICOS), and PD-1 antibodies. The cells were subsequently treated with True Nuclear Transcription Factor Buffer Set (BioLegend) per manufacturer’s guidelines before performing intracellular staining with intracellular markers: anti-FOXP3, Ki-67, and CTLA-4 antibodies (Table S3). The samples were read with BD FACSAria Fusion (BD Bioscience).

### Statistical analysis

Flow cytometry data were analyzed with FlowJo version 10.8.1 (BD Bioscience). Statistical analyses were performed by using GraphPad Prism 9.0 (GraphPad Software, USA). Data are shown as median with interquartile range. For non-paired data, Mann–Whitney *U* test was used to compare between two groups, and Kruskal–Wallis test followed by Dunn’s multiple comparison test was done for multiple comparisons. Statistical analysis of two groups for paired samples was performed by Wilcoxon matched pairs signed-rank test, while Friedman test followed by Dunn’s multiple comparison test was used for more than two groups of paired data. Correlations were determined by Spearman analysis. All data analyses were considered significant for *P < 0.05, **P < 0.01, ***P < 0.001, and ****P < 0.0001.

## RESULTS

### Increased frequencies and proliferation of Tregs in the peripheral blood of dengue patients

**Fig 1:**
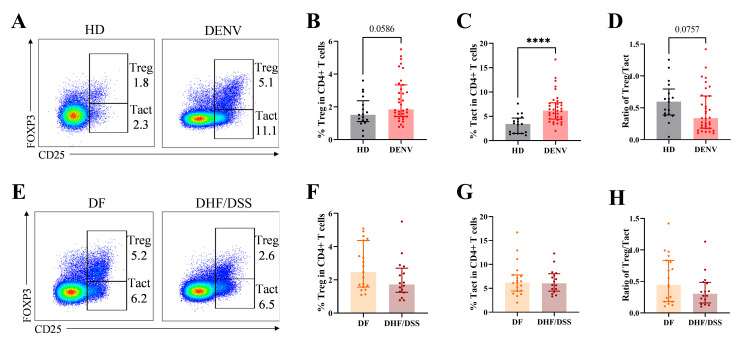
Frequency of Tregs in pediatric dengue patients. (**A**) Representative dot plots of Tregs (CD4^+^CD25^+^FOXP3^+^) and Tacts (CD4^+^CD25^+^FOXP3^−^) in total CD4^+^ T cells from age-matched HD (left) and dengue patients (right). (**B–D**) The frequency of Tregs and Tacts in total CD4^+^ T cells and the ratio of Tregs/Tacts comparing HD with acute DENV-infected patients. (**E**) Representative dot plots of Tregs (CD4^+^CD25^+^FOXP3^+^) and Tacts (CD4^+^CD25^+^FOXP3^−^) in total CD4^+^ T cells from DF (left) and DHF/DSS patients (right). (**F–H**) The frequency of Tregs and Tacts in total CD4^+^ T cells and the ratio of Treg/Tacts comparing DF with DHF/DSS patients. Seventeen age-matched HD, 35 pediatric dengue patients consisting of 19 DF, and 16 DHF/DSS patients were included. The bars indicate median with interquartile range. Mann–Whitney *U* test was used for statistical analysis (*P < 0.05; ****P < 0.0001).

First, we aimed to understand if there were any differences in the frequencies of Tregs (CD4^+^CD25^+^FOXP3^+^) and activated T cells (Tacts: CD4^+^CD25^+^FOXP3^−^) in the blood of patients with acute dengue infection (<4 days after onset of fever; see Table 1) compared to HDs or in patients undergoing classical DF compared to more severe DHF/DSS patients (Fig. S1). Secondary heterotypic infection is an important risk factor for severe disease, probably due to an inappropriately regulated immune response to the secondary infecting serotype. Therefore, we focused our analysis on secondary dengue-infected patients only. There was a tendency for an increase in the frequency of Tregs (*P* = 0.059). The frequency of Tacts was increased in dengue patients compared to healthy controls (*P* < 0.0001) (Fig. 1A through C). This led to a trend toward a decrease in the ratio of Treg over Tacts in DENV-infected patients (*P* = 0.076) (Fig. 1D). When we subdivided the hospitalized dengue cases in either classical DF or DHF/DSS cases, we did not observe any differences in either the frequency of Tregs or Tacts (Fig. 1E through H). Next, we assessed the frequencies of Helios^+^ and Helios^−^ Tregs. HDs and dengue patients showed the same frequency of Helios^+^ and Helios^−^ Tregs (Fig. S2A and B). Moreover, frequencies of Helios^+^ and Helios^−^ Tregs were not different when we categorized dengue patients into mild DF and severe DHF/DSS cases (Fig. S2C and D).

**Fig 2:**
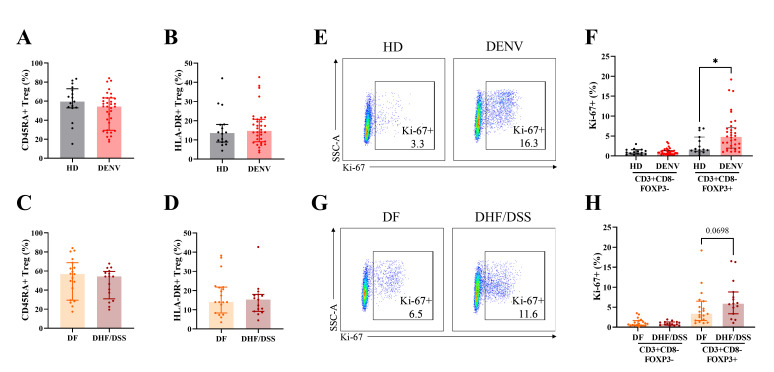
Activation and proliferation state of Tregs from pediatric dengue patients. (**A and B**) The frequencies of CD45RA^+^ Treg and HLA-DR^+^ Treg in HD and dengue patients. (**C and D**) The frequencies of CD45RA^+^ Treg and HLA-DR^+^ Treg in DF and DHF/DSS patients. (**E**) Representative dot plots of the percentages of Ki-67^+^ in CD3^+^CD8^−^FOXP3^+^ in HD (left) and dengue patients (right). (**F**) Summary of the frequencies of Ki-67^+^ in CD3^+^CD8^−^FOXP3^−^ and CD3^+^CD8^−^FOXP3^+^ population. (**G**) Representative dot plots of the percentages of Ki-67^+^ in CD3^+^CD8^−^FOXP3^+^ in DF (left) and DHF/DSS (right). (**H**) Summary of the frequencies of Ki-67^+^ in CD3^+^CD8^−^FOXP3^−^ and CD3^+^CD8^−^FOXP3^+^ population. Seventeen age-matched HD, 35 pediatric dengue patients consisting of 19 DF, and 16 DHF/DSS patients were included. The bars indicate median and interquartile range of the data. Mann–Whitney *U* test was used for statistical analysis (*P < 0.05; ****P < 0.0001).

Next, we evaluated the activation and proliferation state of Tregs in dengue patients and healthy controls. There was no difference in the expression of the naïve/memory markers CD45RA and HLA-DR between dengue patients and healthy controls (Fig. 2A through D). The expression of Ki-67, an early proliferation marker, was increased in Tregs from DENV patients (gated as CD3^+^CD8^−^FOXP3^+^ cells in this panel) compared to Tregs from HD (Fig. 2E and F). This difference was only present in Tregs and not in conventional CD4^+^ T cells (gated as CD3^+^CD8^−^FOXP3^−^). Moreover, proliferation of Treg appeared to be increased mainly in severe dengue patients compared to that in DF patients, even though this difference was not significant (*P* = 0.07; Fig. 2G and H).

Taken together, these data indicated that dengue infection was associated with a modest increase in the frequencies of both Treg and activated T cells and that Treg proliferation was associated with more severe dengue.

### Increase in frequency of Th1-like Tregs in dengue patients

**Fig 3:**
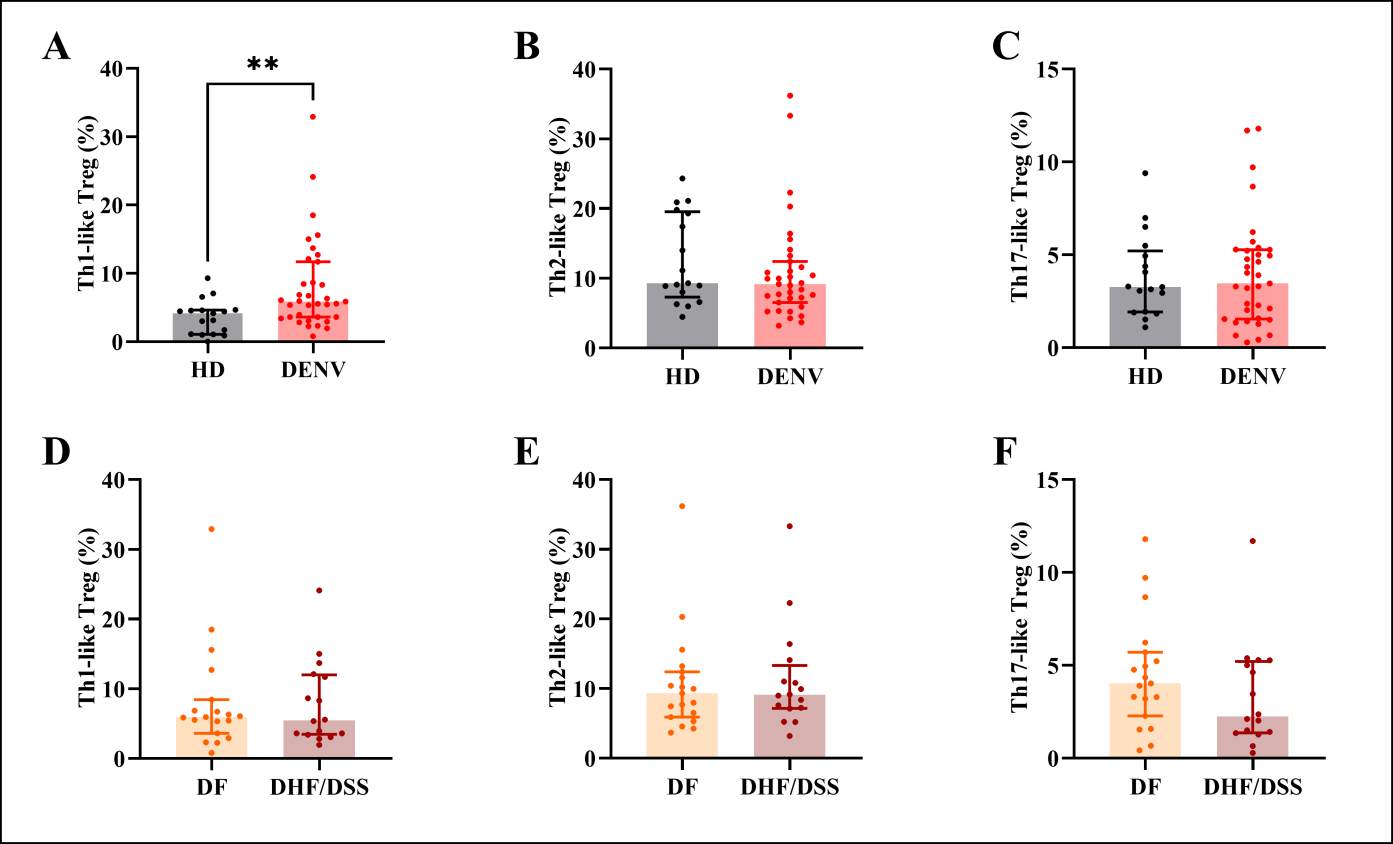
Treg subsets based on chemokine receptors. (**A–C**) Frequency of Th1-like, Th2-like, and Th17-like Treg in HD and dengue patients. (**D–F**) Frequency of Th1-like, Th2-like, and Th17-like Treg in DF and DHF/DSS patients. Seventeen age-matched HD, 35 pediatric dengue patients consisting of 19 DF, and 16 DHF/DSS patients were included. The graphs display median with interquartile range of the data. Mann–Whitney *U* test was used for statistical analysis (**P < 0.01).

Similar to conventional CD4^+^ T cells, Tregs can be skewed to different Th subsets based on their chemokine expression pattern. IFN-γ-producing Th1-like Tregs have been shown to be pro-inflammatory and less suppressive and were increased in autoimmune diseases like multiple sclerosis (MS) or type 1 diabetes (19, 43–45). The frequency of Th1-like Tregs (CXCR3^+^CCR4^−^CCR6^−^ Tregs) was increased in dengue patients compared to HD control (*P* = 0.003; Fig. 3A). However, we found no differences in the frequencies of Th2 (CCR4^+^CXCR3^−^CCR6^−^) or Th17-like Tregs (CCR4^+^CCR6^+^CXCR3^−^) (Fig. 3B and C). Comparing DF to DHF/DSS cases, we did not observe any differences in the frequencies of the different Treg subsets (Fig. 3D through F).

### No difference in functional markers in Tregs from mild or severe dengue patients

**Fig 4:**
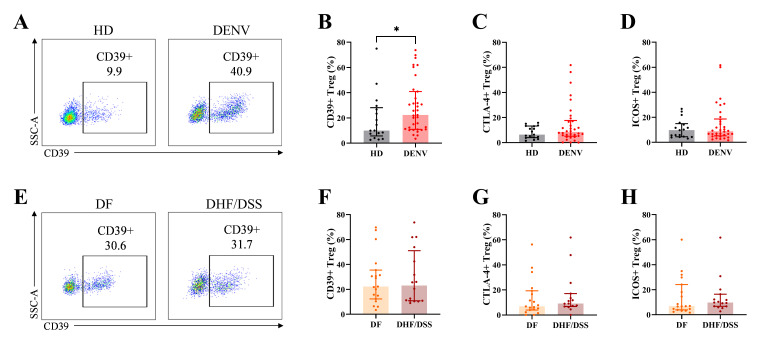
Expression of functional markers in Tregs. (**A**) Representative dot plots of CD39^+^ Tregs in HD (left) and dengue patients (right). (**B–D**) Frequency of CD39^+^ Tregs, CTLA-4^+^ Tregs and ICOS^+^ Tregs in HD and dengue patients. (**E**) Representative dot plots of CD39^+^ Tregs in DF (left) and DHF/DSS patients (right). (**F–H**) Frequency of CD39^+^ Tregs, CTLA-4^+^ Tregs and ICOS^+^ Tregs in DF and DHF/DSS patients. Seventeen age-matched HD, 35 pediatric dengue patients consisting of 19 DF, and 16 DHF/DSS patients were included. The graphs display median and interquartile range of the data. Mann–Whitney *U* test was used for statistical analysis (*P < 0.05).

Tregs execute their suppressive function via direct cell–cell contact mediated by molecules such as ICOS or CTLA-4 or by disruption of metabolic pathways mediated by CD39 and CD73 (46). Therefore, we evaluated the expression of these functional markers in Tregs from dengue patients. Whereas no difference was observed for CTLA-4 or ICOS expression, we noticed an upregulation of CD39 expression in dengue patients compared to healthy control (*P* = 0.038; Fig. 4A through D). No differences in the expression of the functional markers were observed between classical DF and DHF/DSS cases (Fig. 4E through H). These data suggest that for the markers that were evaluated in the current study, Tregs from mild and severe dengue patients have the same characteristics in the acute phase of infection.

### Tregs from severe dengue patients produce higher amounts of pro-inflammatory cytokines

**Fig 5:**
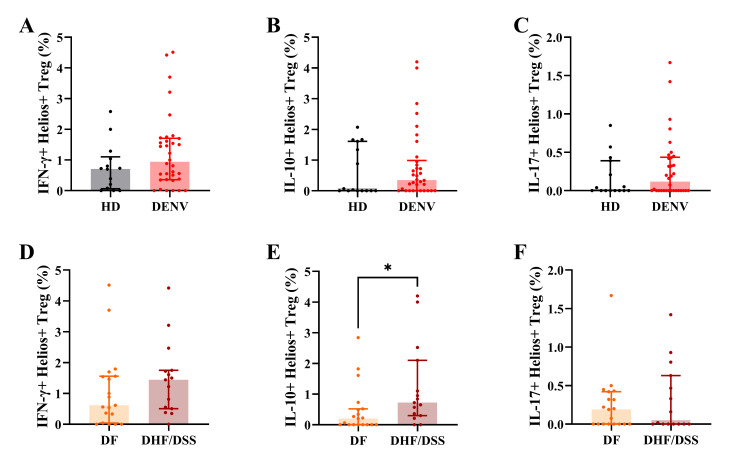
Cytokine production in Helios^+^ Tregs. (**A–C**) Frequency of IFN-γ^+^Helios^+^ Tregs, IL-10^+^Helios^+^ Tregs, and IL-17^+^Helios^+^ Tregs in HD and dengue patients. (**D–F**) Frequency of IFN-γ^+^Helios^+^ Tregs, IL-10^+^Helios^+^ Tregs, and IL-17^+^Helios^+^ Tregs in DF and DHF/DSS patients. Fifteen age-matched HD, 34 pediatric dengue cases consisting of 19 DF, and 15 DHF/DSS patients were included. The graphs show median with interquartile range of the data. Mann–Whitney *U* test was used for statistical analysis (*P < 0.05).

In addition to performing their suppressive function via contact-mediated suppression, Tregs also produce anti-inflammatory cytokines such as IL-10, IL-35, and transforming growth factor beta (TGF-β) (46). However, under inflammatory conditions, Tregs can be skewed to more Th1-like Tregs, as seen in Fig. 3A, which can produce pro-inflammatory cytokines such as IFN-γ (47). Therefore, we assessed cytokine production by Helios^+^ and Helios^−^ Tregs by intracellular cytokine staining after PMA/ionomycin stimulation. Helios^+^ Tregs from dengue patients seem to produce more IFN-γ and IL-10 compared to HD Helios^+^ Tregs, even though this did not reach statistical significance (Fig. 5A through C; Fig. S3). Interestingly, Helios^+^ Tregs from severe dengue patients tended to produce more IFN-γ and IL-10 compared to DF patients (*P* = 0.255, *P* = 0.017; Fig. 5D through F). No differences in cytokine production were observed in the Helios^−^ Treg compartment (Fig. S4).

### *In vitro* infection of PBMC with DENV modulates expression of functional markers on Tregs

Next, to understand how DENV infection could affect the Treg phenotype, we infected HD PBMC with DENV and evaluated the Treg phenotype (Fig. S5 and S6). As expected, monocytes were infected at MOI one as observed after staining with the pan-flavivirus anti-E antibody clone 4G2 (Fig. S7). The infection increased significantly from 1.7% for MOI 0.1 to 13.1% for MOI 1 (*P* = 0.002), while no infected monocytes were detected in the negative controls (Fig. S7D). As expected, CD3^+^ T cells were not infected with DENV (Fig. S7C). Therefore, in this *in vitro* model, all effects observed on Tregs are the consequence of DENV infection on other PBMC subsets, mainly monocytes. We evaluated the expression of activation markers and functional markers on Tregs 3 days after *in vitro* infection (Fig. S5 and S6). We observed an increase in the expression of functional markers such as CTLA-4 and PD-1 in Tregs from DENV-infected cultures, especially at high titer of virus infection, compared to mock-infected conditions (*P* = 0.022, *P* = 0.022; Fig. S5B through G). In contrast to our *ex vivo* findings, *in vitro* DENV infection resulted in a small but significant decrease on the percentage of Ki-67^+^ Tregs (*P* = 0.042) (Fig. S5D). Taken together, *in vitro* infection of PBMC by DENV resulted in an altered Treg phenotype, which did not recapitulate the *ex vivo* observations, likely due to different kinetics and conditions of the *in vitro* model system.

### Tregs from dengue patients have decreased suppressive capacity

**Fig 6:**
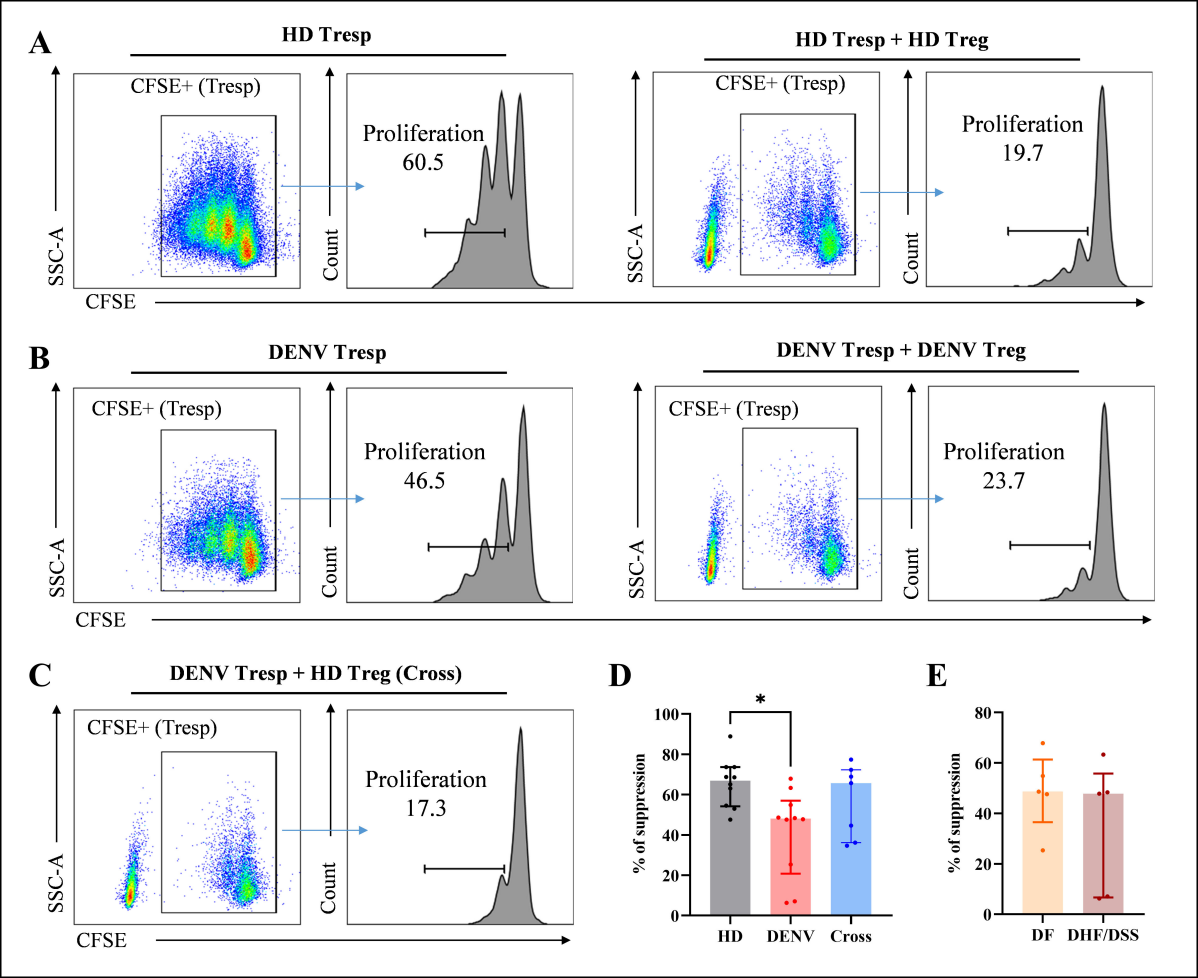
*In vitro* suppression assay measuring Treg functionality. (**A–C**) Representative plots showing gating strategy for cell proliferation in HD samples, dengue patient samples, and a cross-culture between Tregs from HD and Tresps from DENV patients. A total of 10,000 Tresp cells were used for positive control, and 5,000 Treg cells with 5,000 Tresp cells were used for co-culture and cross-culture with 1:1 ratio to Treg suppressive inspector. (**D and E**) Summary of percentages of each suppression assay show low suppressive function of Tregs in pediatric dengue patients compared to age-matched HD (HD: *n* = 10; DENV: *n* = 10; DF: *n* = 5; and DHF/DSS: *n* = 5). Bars indicate median with interquartile range. Kruskal–Wallis test followed by Dunn’s multiple comparison test was used for multiple comparisons (**D**), and Mann–Whitney *U* test was used for two groups (**E**).

Finally, we assessed the suppressive capacity of Tregs by *in vitro* suppression assays co-culturing sorted Tregs (CD4^+^CD25^+^CD127 low) and T responder (Tresp, CD4^+^CD25^−^) cells in the presence of CD2/CD3/CD28 stimulation (Fig. S8; Fig. 6A through C). When we compared the capacity of Tregs isolated from HD or DENV patients to suppress the proliferation of autologous Tresp cells, we observed that Tregs from DENV patients showed less suppressive capacity compared to Tregs from HD (*P* = 0.024; Fig. 6D). This effect could be due to either a defect in Treg capacity or a hyperactivation of Tresp, which renders the Tresp unresponsive to suppression (48, 49). Therefore, we performed cross-culture experiments where Tregs from HD were incubated with Tresp from DENV patients. Here, we observed no statistical difference in Treg function whether HD Tresp or DENV Tresp is used in the assay (Fig. 6D). However, in these cross-experiments, three samples did show decreased suppression by HD Tregs, indicating that the effect might be partially driven by a Treg defect and partly by an inability of the Tresp cells to be suppressed. Unfortunately, due to the small sample volumes obtained from pediatric dengue patients, cross-culture assays with Treg from DENV patients could not be performed. Finally, we compared the suppression capacity of Tregs obtained from DF or DHF/DSS patients in the autologous suppression assay and noticed a similar low percentage of suppression in both patient groups indicating that Tregs from both mild and severe dengue patients exhibited reduced capacity to suppress Tresp proliferation (Fig. 6E). Taken together, this *in vitro* functional analysis indicated that Tregs obtained from DENV-infected patients in the acute phase of infection are less effective in suppressing Tresp proliferation compared to Tregs obtained from HD.

## DISCUSSION

In this study, we investigated if Tregs have an altered phenotype and function in hospitalized dengue-infected patients. Tregs are important in maintaining immune homeostasis and contribute to the contraction phase after the clearance of infection via several mechanisms such as direct inhibition of effector T cells, skewing of dendritic cell maturation or cytokine production (19, 20). During the acute phase of viral infection, Tregs can play a dual role in protection or pathology. On one hand, a suppressive Treg response could be detrimental early in the disease course by inhibiting antigen-specific T-cell activation and function and by inhibiting Treg cell death, thereby interfering with efficient and timely viral clearance and (29, 30, 50). On the other hand, Tregs can suppress proinflammatory signals which may protect from cytokine storm and tissue injury (30).

We found that frequencies of Tregs are increased in acute dengue patients compared to age-matched healthy children, which is in accordance with previous findings (31, 32). However, also the frequency of activated T cells increased, leading to a decreased ratio of Tregs over-activated T cells. The rapid expansion of Tregs observed in the acute phase of disease in our patient cohort could be due to the fact that we selected secondary infected patients for this analysis; hence, serotype cross-reactive memory T cells, including Tregs, will respond rapidly after infection (51). This is corroborated by the finding that the frequency of Ki-67^+^ Tregs is increased in dengue patients compared to healthy controls (Fig. S9).

An association between frequencies of Tregs and dengue disease severity is less clear. Some studies showed that Tregs were expanded in patients with mild dengue, but not in patients with severe dengue (32, 36). However, a recent study showed increased gene expression of *FOXP3* in DHF compared to DF patients (34). Interestingly, the frequency of Tregs was increased in acute dengue-infected patients who progressed to severe dengue compared to patients who did not develop severe complications (37, 38). Along the same line, in this study, we found that Tregs from severe dengue patients seemed to proliferate more compared to Tregs from classical DF patients, even though our results did not reach significance.

Memory-like Tregs may adapt their function to the nature of the immune response and can express the master transcription factor of the T effector cell population that they suppress. For example, they can express T-bet in type 1 inflammation (25), they adopt the chemokine receptor expression profile of Th1 cells to allow them to migrate to the site of Th1 inflammation, and they can produce IFN-γ (25). Here, IFN-γ can act in an autocrine/paracrine manner to maintain the Th1-like phenotype (52). Indeed, IFN-γ induction of Th1-like Tregs controls antiviral responses, at least in mouse models (29). Our detailed phenotypic analysis showed an increase in the frequency of Th1-like Tregs (CXCR3^+^CCR4^−^CCR6^−^ Tregs) in all dengue patients, irrespective of disease severity. In concurrence, we observe that Helios^+^ Tregs from dengue patients tended to produce more IFN-γ, compared to healthy controls which seemed further increased in severe dengue patients.

In terms of functional markers, Tregs expressed the same levels of ICOS and CTLA-4 in dengue patients compared to healthy controls, and no differences were noted with respect to disease severity, in accordance with another study in a cohort from Sri Lanka (31). However, other studies have shown that polymorphisms in the genes for CTLA-4 are associated with DHF and higher frequencies of Tregs from dengue progressors expressed CTLA-4, indicating that a closer investigation of the role of CTLA-4 in dengue pathogenesis is warranted (37, 38, 53).

CD39 (ectonucleoside triphosphate diphosphohydrolase 1) which converts ATP into adenosine diphosphate is the rate-limiting enzyme in the generation of immune-suppressive adenosine and is a marker of highly active and suppressive Tregs (54, 55). The presence of increased frequencies of CD39^+^ Tregs has been associated to progression of both HBV and HIV infections (56–58). Our findings show increased frequencies of CD39^+^ Tregs in dengue patients; however, we did not observe any differences comparing mild and severe patients.

Whereas *in vitro* infection of PBMC with DENV induced functional markers such as CTLA-4, ICOS, and PD-1, no differences in the expression of these markers were observed in Tregs phenotyped directly *ex vivo* from DENV-infected patients. This could be due to the fact that the *in vitro* system does not reflect the migration of Tregs and their maturation in other compartments rather than the blood *in vivo*. In addition, we did not evaluate the effect of antibody-mediated infection of monocytes in our *in vitro* culture. It is known that infection mediated by DENV-immune complexes and FcγR engagement leads to altered cytokine production by the infected monocytes and dendritic cells which could have effects on Treg maturation *in vivo* (59–61).

Activated Tregs in the blood might migrate to target tissues of the disease, such as the liver. In addition, Tregs will maturate and gain functional potency in the lymph nodes, which also contain a high amount of several dendritic cell subsets, which can be infected by DENV (59, 62, 63). Therefore, analysis of the blood compartment might not reflect what is occurring in key tissue sites of disease. In liver biopsies of DHF patients, FOXP3 was noticeably absent, while pro-inflammatory markers such as TLR2, iNOS, IL-6, IL-18, TGF-β, and granzyme B were highly expressed (64). The authors hypothesized that the expression of FOXP3 could be reduced by the presence of these pro-inflammatory cytokines produced by innate immune cells in severe dengue (64).

Dengue is endemic in tropical and subtropical regions of the world. Within endemic settings, dengue is mostly occurring in younger children; hence, obtaining sufficient blood to perform *in vitro* functional assays is challenging. A previous study demonstrated that Tregs are able to suppress the proliferation of Tresp at a similar degree in the acute phase and after recovery, although no comparisons with healthy controls were made and sample size was limited (32). Moreover, Tregs isolated during the acute phase of DENV infection were able to suppress IFN-γ production in Tresp and TNF production of in monocytes after *in vitro* co-culture, two cytokines with an important vasoactive function (32). However, we showed that Tregs obtained from dengue pediatric cases are less able to suppress proliferation of Tresp in an *in vitro* proliferation assay. This was not due to hyperactivation of Tresp obtained from DENV-infected cases, which renders the Tresp unresponsive to suppression (49) as Tregs from healthy controls could suppress proliferation of Tresp obtained from dengue patients.

Although beneficial during viral infection, strong Th1 responses must be counterbalanced to prevent unwanted tissue destruction and immunopathology. FOXP3^+^ Tregs are essential for the proper regulation of Th1 responses *in vivo*, and loss of Tregs results in uncontrolled Th1 responses, further demonstrating the important and non-redundant function of Tregs in dampening type 1 inflammation (65). Tregs could contribute to dengue immunopathogenesis via different mechanisms. A recent study investigating both the B- and T-cell compartments in a cohort of Singaporean patients revealed that frequencies of Tregs correlated positively with proliferating plasmablasts (Ki-67^+^) during acute disease, whereas CD38^+^CCR7^−^ Tregs (effector memory-like Tregs with high suppressive function) correlated negatively with PD-1 expressing plasmablasts indicating a potential regulatory role for Tregs in plasmablast formation (35). We found that Tregs from severe dengue patients produced higher amounts of IL-10 after PMA/ionomycin stimulation, a cytokine that has been implicated in dengue immunopathogenesis and which can be found at increased concentrations in patients with severe dengue (9, 10, 66–68). Induction of different cytokines is time- and stimulation-dependent (69, 70). One limitation of the study is that we could only include one type of stimulation and one time point, given the limited volume of blood available for the study.

Dengue is a highly dynamic disease with a fast progression toward severe disease. Therefore, the balance of beneficial versus detrimental effects of different subsets of Tregs could shift during the course of the immune response. Additional analysis of the dynamic changes in Treg subsets and functions would be informative yet difficult to accomplish in dengue-endemic regions (37, 38).

In conclusion, we showed that Tregs from DENV-infected patients are expanded but have less suppressive capacity compared to Tregs obtained from age-matched HDs and are skewed to a Th1-like Treg phenotype with increased production of cytokines after stimulation. Together with the observed increase of activated T cells during infection, these data suggest that Tregs fail to resolve inflammation and hence contribute to dengue immunopathology. In conclusion, this work identified the modulation of Treg function as a possible therapeutic strategy in severe dengue.

## SUPPLEMENTAL MATERIAL

Supplemental material mbio.00063-24-s0001.docx Supplemental tables and figures.

ASM does not own the copyrights to Supplemental Material that may be linked to, or accessed through, an article. The authors have granted ASM a non-exclusive, world-wide license to publish the Supplemental Material files. Please contact the corresponding author directly for reuse.
