# Supplementary material for: Increased frequencies of highly activated regulatory T cells skewed to a T helper 1-like phenotype with reduced suppressive capacity in dengue patients
Source: mBio. 2024 May 16;15(6):e00063-24. doi: 10.1128/mbio.00063-24 (PMC11237415; doi:10.1128/mbio.00063-24)
Supplement: Supplemental material — Supplemental tables and figures. [file mbio.00063-24-s0001.docx]

**Supplemental figures**

**Supplemental figure 1: Gating strategy to assess Treg phenotype.** (A) Evaluation of the expression of activation, migration and functional markers (Treg: CD4+CD25+FOXP3+ and Tact: CD4+CD25+FOXP3-) and (B) Evaluation of Ki-67 expression and cytokine production (Tregs: CD3+CD8-FOXP3+, non-Tregs: CD3+CD8-FOXP3-)

**Supplemental figure 2:** **Evaluation of Helios+ and Helios- Treg subsets.** (A, C) Frequency of thymic Tregs (CD3+CD8-FOXP3+Helios+) and (B, D) peripheral Tregs (CD3+CD8-FOXP3+Helios-). 15 age-matched HD, 34 pediatric dengue patients consisting of 19 DF and 15 DHF/DSS patients were included. Bars indicate median with interquartile range. Mann-Whitney test was used for statistical analysis.

**Supplemental figure 3:** **Representative dot plots of cytokine production in Helios+ Tregs.** (A, B, C) IFN-γ, IL-10 and IL-17 production in age-matched healthy controls. (D, E, F) IFN-γ, IL-10 and IL-17 production in pediatric dengue patients, respectively.

**Supplemental figure 4:** **Cytokine production in Helios- Tregs.** (A-C) Frequency of IFN-γ+ Helios- Tregs, IL-10+ Helios- Tregs, and IL-17+ Helios- Tregs in HD and dengue patients. (D-F) Frequency of IFN-γ+ Helios- Tregs, IL-10+ Helios- Tregs, and IL-17+ Helios- Tregs in DF and DHF/DSS patients. 15 age-matched HD, 34 pediatric dengue cases consisting of 19 DF and 15 DHF/DSS patients were included. The graphs show median with interquartile range of the data. Mann-Whitney test was used for statistical analysis (*p < 0.05).

**Supplemental figure 5:** **Expression of functional markers 3 days after *in vitro* DENV infection.** (A) Summary of *in vitro* DENV infection of adult HD PBMCs (n=10). (B) Frequencies of Tregs (CD3+CD4+CD25+CD127lowFOXP3+) in CD3+CD4+ population after mock-infection or infection with DENV at MOI 0.1 or MOI 1. (C-G) Frequencies of HLA-DR+, Ki-67+, CTLA-4+, ICOS+, and PD-1+ Tregs in PBMC cultures after *in vitro* DENV infection with either MOI 0.1 or MOI 1, compared to mock-infected cultures (control). Bars indicate median and interquartile range. Friedman test followed by Dunn’s multiple comparison test was used for statistical analysis. *p < 0.05, **p < 0.01.

**Supplemental figure 6: Representative gating strategy to assess the expression of activation markers after *in vitro* DENV infection**. Tregs were gated as CD3+CD4+CD25CD127lowFOXP3+.

**Supplemental figure 7: Evaluation of DENV infection after *in vitro* culture.** (A) Representative gating strategy for monocytes and T cells 24h after *in vitro* infection. (B and C) Representative dot plot of 4G2+ cells in monocytes and T cells, respectively. (D) Percentage of infected monocytes (n=10). Bars indicate median with interquartile range. Wilcoxon matched-pairs signed rank test was used for statistical analysis (D).

**Supplemental figure 8:** **Gating strategy for cell sorting.** (A) Representative dot plots of gating strategy for Tresp (CD4+CD25-) and Tregs (CD4+CD25+CD127low) used for the *in vitro* suppression assay. (B and C) Representative dot plots of sorted Tresp and Treg cells, respectively.

**Supplemental figure 9:** **Graphical abstract of the study.**

**Supplemental tables**

**Supplemental table 1**: Panels for characterizing Treg subpopulations by flow cytometry

| **Panel** | **BV510** | **PerCP-cy5.5** | **APC** | **PE** | **BV421** | **FITC** | **PE-cy7** |
| --- | --- | --- | --- | --- | --- | --- | --- |
| 1 | DUMP* | CD4 | CD25 | *FOXP3* | CD45RA | CD39 |  |
| 2 | DUMP* | CD4 | CD25 | *FOXP3* | CXCR3 | CCR6 | CCR4 |
| 3 | DUMP* | CD4 | CD25 | *FOXP3* | *CTLA-4* | HLA-DR | ICOS |

*Zombie Aqua, CD14, CD19, and CD56; *Intracellular staining*

**Supplemental table 2**: Cytokine production panel

| **Panel** | **BV510** | **PerCP-cy5.5** | **APC** | **PE** | **APC-fire** | **BV421** | **FITC** | **PE-cy7** |
| --- | --- | --- | --- | --- | --- | --- | --- | --- |
| 4 | DUMP* | *IFN-γ* | *IL-17A* | *FOXP3* | CD3 | *Ki-67* | *Helios* | *IL-10* |

*Zombie Aqua, CD8a, CD14, CD19, and CD56, *Intracellular staining*

**Supplemental table 3:** Detailed list of all antibodies used in this study

| **Channel** | **Name** | **Clone** | **Supplier** | **Catalog N.** |
| --- | --- | --- | --- | --- |
| PerCP-Cy5.5 | CD4 | OKT4 | Biolegend | 317428 |
| APC | CD25 | BC96 | Biolegend | 302610 |
| APC-Fire | CD127 | A019D5 | Biolegend | 351350 |
| PE | FOXP3 | 259D | Biolegend | 320208 |
| FITC | CD39 | A1 | Biolegend | 328206 |
| BV421 | CD45RA | HI100 | Biolegend | 304130 |
| FITC | CCR6 | G034E3 | Biolegend | 353412 |
| PE-Cy7 | CCR4 | L291H4 | Biolegend | 359410 |
| BV421 | CXCR3 | G025H7 | Biolegend | 353716 |
| AF488 | HLA-DR | L243 | Biolegend | 307620 |
| PE-Cy7 | ICOS | C398.4A | Biolegend | 313520 |
| BV421 | CTLA-4 | BNI3 | Biolegend | 369606 |
| BV510 | CD14 | 63D3 | Biolegend | 367124 |
| BV510 | CD19 | HIB19 | Biolegend | 302242 |
| BV510 | CD56 | 5.1H11 | Biolegend | 362534 |
| BV510 | CD8a | RPA-T8 | Biolegend | 301048 |
| APC-fire | CD3 | OKT3 | Biolegend | 317351 |
| FITC | Helios | 22F6 | Biolegend | 137214 |
| BV421 | Ki-67 | Ki-67 | Biolegend | 350506 |
| PerCP-Cy5.5 | IFN-γ | B27 | Biolegend | 506528 |
| PE-Cy7 | IL-10 | JES3-9D7 | Biolegend | 501420 |
| APC | IL-17A | BL168 | Biolegend | 512334 |
| PE-Cy7 | CD127 | A019D5 | Biolegend | 351320 |
| BUV395 | CD3 | SK7 | BD Bioscience | 564001 |
| BUV496 | CD4 | SK3 | BD Bioscience | 612936 |
| BUV737 | CD45RA | HI100 | BD Bioscience | 612846 |
| BV605 | CD194 (CCR4) | L291H4 | Biolegend | 359418 |
| BV650 | CD279 (PD-1) | EH12.2H7 | Biolegend | 329950 |
| BV711 | CD183 (CXCR3) | G025H7 | Biolegend | 353732 |
| BV786 | CD39 | A1 | Biolegend | 328240 |
| BB515 | CD196 (CCR6) | 11A9 | BD Bioscience | 564479 |
| PE | FOXP3 | 150D | Biolegend | 320008 |
| PE/Dazzle | CD278 (ICOS) | C398.4A | Biolegend | 313532 |
| PerCP/Cy5.5 | HLA-DR | L243 | Biolegend | 307629 |
| APC | CD152 (CTLA-4) | BNI3 | Biolegend | 369612 |
| APC-R700 | CD25 | 2A3 | BD Bioscience | 565106 |
| APC-Fire | CD127 (IL-7Rα) | A019D5 | Biolegend | 351350 |
| PerCP-Cy5.5 | CD3 | L243 | Biolegend | 307630 |
| APC | CD14 | BNI3 | Biolegend | 369612 |
| BV421 | CD56 | 5.1H11 | Biolegend | 362552 |
